# Mechanism of Introduction of Exogenous Genes into Cultured Cells Using DEAE-Dextran-MMA Graft Copolymer as Non-Viral Gene Carrier

**DOI:** 10.3390/molecules14072669

**Published:** 2009-07-23

**Authors:** Yuki Eshita, Junko Higashihara, Masayasu Onishi, Masaaki Mizuno, Jun Yoshida, Tomohiko Takasaki, Naoji Kubota, Yasuhiko Onishi

**Affiliations:** 1 Department of Infectious Disease Control, Faculty of Medicine, Oita University, 1-1 Idaigaoka, Hasama-machi, Yufu-shi, Oita Prefecture 879-5593, Japan; 2 Ryujyu Science Corporation, 39-4 Kosora-cho, Seto-shi, Aichi Prefecture 489-0842, Japan;; 3 The Center for Genetic and Regenerative Medicine, Nagoya University Graduate School of Medicine, 65 Tsuruma-cho, Showa-ku, Nagoya-shi, Aichi Prefecture 466-8550, Japan; E-mail: mmizuno@med.nagoya-u.ac.jp (M.M.); 4 Department of Neurosurgery, Nagoya University Graduate School of Medicine (65 Tsuruma-cho, Showa-ku, Nagoya-shi, Aichi Prefecture 466-8550, Japan; E-mail: jyoshida@med.nagoya-u.ac.jp (J.Y.); 5 Department of Virology 1, National Institute of Infectious Diseases, 1-23-1 Toyama, Shinjyuku-ku, Tokyo 162-8640, Japan; E-mail: takasaki@nih.go.jp (T.T.); 6 Department of Chemistry, Faculty of Medicine, Oita University, 1-1 Idaigaoka, Hasama-machi, Yufu-shi, Oita Prefecture 879-5593, Japan; E-mail: nkubota@med.oita-u.ac.jp (N.K.)

**Keywords:** transfection efficiency, DEAE-dextran-MMA graft copolymer, non-viral gene carrier, exogenous genes

## Abstract

Comparative investigations were carried out regarding the efficiency of introduction of exogenous genes into cultured cells using a cationic polysaccharide DEAE-dextran-MMA (methyl methacrylate ester) graft copolymer (2-diethylaminoethyl-dextran-methyl methacrylate graft copolymer; DDMC) as a nonviral carrier for gene introduction. The results confirmed that the gene introduction efficiency was improved with DDMC relative to DEAE-dextran. Comparative investigations were carried out using various concentrations of DDMC and DNA in the introduction of DNA encoding luciferase (pGL3 control vector; Promega) into COS-7 cells derived from African green monkey kidney cells. The complex formation reaction is thought to be directly proportional to the transformation rate, but the complex formation reaction between DDMC and DNA is significantly influenced by hydrophobic bonding strength along with hydrogen bonding strength and Coulomb forces due to the hydrophobicity of the grafted MMA sections. It is thought that the reaction is a Michaelis-Menten type complex formation reaction described by the following equation: Complex amount = K1 (DNA concentration)(DDMC concentration). In support of this equation, it was confirmed that the amount of formed complex was proportional to the RLU value.

## Introduction

The development of gene delivery systems is an important area in the field of genetic engineering [1]. A constituent element involves the transport of genes, which requires a transport vehicle referred to as a vector. Vectors include viral “shells” or lipid spheres (liposomes) having properties whereby they are incorporated into host cells. Viral vectors employ a viral shell and part of the viral genome, and the danger of pathogenicity or immunogenicity has thus been highlighted. Liposome vectors are completely artificial and are produced by introducing genes into microspheres that have a lipid bilayer structure similar to that of a cell membrane. However, sterilization by autoclaving is impossible due to their instability at high temperatures. On the other hand, favorable results regarding efficiency have been indicated with commercial cationic lipid micelle transfection reagents. However, these reagents also cannot be sterilized by autoclaving and are thus not amenable to mainstream use as non-viral gene introduction carriers. Electrophoresis and microinjection methods are also examples of electrical and physical methods, but they require special devices and technologies.

Cationic polymers are man-made materials and are thus expected to be stable when heated [2]. Although these compounds have problems with cytotoxicity and low transformation rates, they have a history of use as nonviral vectors, and desirable DEAE-dextrans (2-diethylaminoethyl-dextran) are currently being closely investigated because they can be sterilized [3,4]. Copolymers formed by graft polymerization of methyl methacrylate (MMA) with DEAE-dextran have hydrophilic and hydrophobic regions and are known to be desirable non-viral vectors due to their high transformation efficiency [5,6,7]. Thus, complexes of DNA and DEAE-dextran-MMA graft copolymer (DDMC) produced by modification of DEAE-dextran has been reported to have superior transformation efficiency of 50× or more relative to DEAE-dextran in terms of transformation efficiency in various types of cell [8]. However, details concerning the mechanism are uncertain. By investigating the incorporation of DNA in cells using quantitative means or visual imaging, it would then be possible to clarify this mechanism and to design gene delivery systems at the molecular level. This report presents the results of comparative investigations along these lines regarding the transfection efficiency of DDMC relative to unmodified DEAE-dextran using COS-7 cells and DNA encoding luciferase-expressing genes.

## Results and Discussion

### Transfection into COS-7 Cells

**Figure 1:**
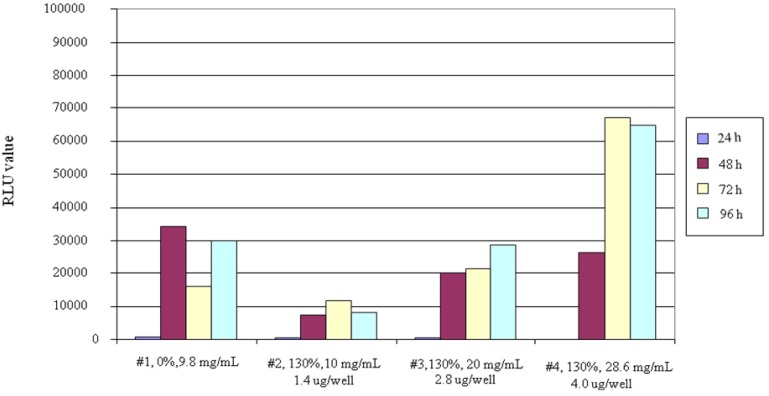
Transfection of COS-7 cells with sample 1 of DEAE-dextran and DEAE-dextran-MMA graft copolymer. The grafting rate is 130% for sample 2 at 10 mg/mL, sample 3 at 20 mg/mL, and sample 4 at 28.6 mg/mL.

We used 96-well microtiter plates, and the methods for transfecting a complex by pGL3-Control Vector DNA and carrier into COS-7 cells were investigated. The results are shown in Figure 1. When comparing the transfection efficiency with DDMC (graft ratio 130%) and DEAE-dextran (graft ratio 0%), the RLU values for DDMC at a concentration of 10.0 mg/mL were lower than for DEAE-dextran, but the efficiency increased in a concentration-dependent manner as DDMC concentration was increased. At a concentration of 20.0 mg/mL, the values were approximately equivalent, but the value was2× higher at a concentration of 28.6 mg/mL. The fact that the efficiency increased in a concentration-dependent manner may be due to an increase in transfection efficiency resulting from low DDMC cell toxicity for their increases in the complex formed with DNA.

### Charge Ratio (P/N ratio)

When considering transfection efficiency, the charge ratio (P/N) of each sample is an important value as well as the concentration. It is thus necessary to make the P/N values equivalent when comparing RLU values for DEAE-dextran and DDMC. When investigating related P/N ratios, for example, the percentage of nitrogen in DEAE-dextran was 3.3%. In addition, the percentage phosphorus in the DNA was about 5.33%. The P/N values shown in Figure 1 were thus obtained.

With regard to dependence of the amount of transferred DNA on P/N ratio (Table 1), it would appear that a comparison can be made of the RLU values with DEAE-dextran (graft ratio 0%) and DDMC (graft ratio 130%) at the respective sample P/N values. In other words, a P/N value of 0.021 is given for DDMC (graft ratio 130%) at a concentration of 28.6 mg/mL, and based on the fact that the P/N value for DEAE-dextran is nearly the same at 0.026, it was concluded that the charge ratios are approximately equivalent. When actually comparing transfection efficiency based on the two RLU values, the RLU value at a DDMC concentration of 28.6 mg/mL is about 2× higher, and this is thought to be due to micelle micro-formation resulting from the hydrophilic-hydrophobic microseparated domain of DDMC.

**Table 1:** Charge ratios (P/N) of DEAE-dextran-MMA graft copolymer (DDMC) and DEAE-dextran to DNA.

	P/N ratio
DEAE-dextran (grafting rate 0%)	0.026
DEAE-dextran-MMA graft copolymer (grafting rate 130%)	
DDMC 28.6 mg/mL	0.021
DDMC 20.0 mg/mL	0.030
DDMC 10.0 mg/mL	0.060

In transfection testing carried out according to the procedure of this report, the transfection efficiency of not only DDMC, but also DEAE-dextran (graft ratio 0%) gave high RLU values relative to the commercially-available product PolyFect (QIAGEN) [9], a result that was obtained in preliminary testing using COS-7 cells. This suggests that there is a fairly large variation in efficiency depending on transfection conditions such as reagent amount etc.

### Expression Time

When comparing the luciferase protein expression times for DDMC and DEAE-dextran in COS-7 cells, though luciferase activity in DDMC at 24 h after transfection seems to be low as well as DEAE-dextran (graft ratio 0%), there was almost no expression especially with DDMC (graft ratio 130%, 28.6 mg/mL). Protein expression with DDMC was confirmed to be extremely high after 48 h. With DDMC (graft ratio 130%), there was almost low expression after 24 h with COS-7 cells. However, there was a trend towards higher RLU values based on protein expression amount when observed over time at 48, 72 and 96 h, as indicated (Figure 1). This is thought to be due to the fact that the DDMC-DNA complex is comparatively stable, and thus a long period of time is required for transport into the cell nucleus, release of the DNA and expression.

The difference in expression times for DEAE-dextran and DDMC was discussed above, but it was determined that the optimal expression time with DDMC was 72 h, based on RLU values. The maximum RLU value of DDMC was seen at 72 h (Figure 1). When each sample corresponds in time in accordance with this reasoning, it is thought that 72 h is favorable based on a comparison of RLU values. Otherwise, a comparison is made at the respective maximum values.

When comparing transfection efficiency for DEAE-dextran (graft ratio 0%) and DDMC in transfection experiments carried out using HEK293 cells, remarkable results, to have an amphiphilic domain in order to form a polymer micelle, were obtained with DDMC (graft ratio 130%) [5,6,7,8,9].

However, in the experiments, concentrations were limited to 28.6 mg/mL and 20.0 mg/mL, and the expression with samples having a concentration of 10.0 mg/mL was lower than with DEAE-dextran. This is conjectured to be due to the absolute amount of transported DNA with this experimental system and not due to problems with DEAE-dextran cell toxicity or cellular toxicity with respect to DDMC concentration.

In addition, considering the optimal value for expression time discussed above, it was thought there is the different mechanism of transfection between DEAE-dextran and DDMC. This is indicated by the fact that almost no luciferase protein is expressed in COS-7 cells at 24 h after transfection especially with DDMC (graft ratio 130%, 28.6 mg/mL), in contrast to DEAE-dextran (graft ratio 0%). It was thought that DNA condensation may play an important role in transfection efficiency [10,11] and that the dissociation conditions of the complex by DNA and DDMC when introduced into the nucleus also may differ.

### Complex Formation

One objective purpose of using DDMC is that a stable complex with DNA is formed. Specifically, complex formation between DEAE-dextran and DNA is not so stable, and decomposition by intracellular dextransucrase should be thought to occur after transport into the cell. For this reason, DNase protection is decreased, thus decreasing transfection efficiency. In addition, the DEAE-dextran concentration cannot be increased due to cellular toxicity. This is one of the reasons for using DDMC that has been freshly developed. With DDMC, vinyl monomer is graft-polymerized onto DEAE-dextran in order to stabilize the complex with DNA. It is thought that an effect of this stabilization is the delay of luciferase expression [11]. 

### DNase Protective Activity

**Figure 2:**
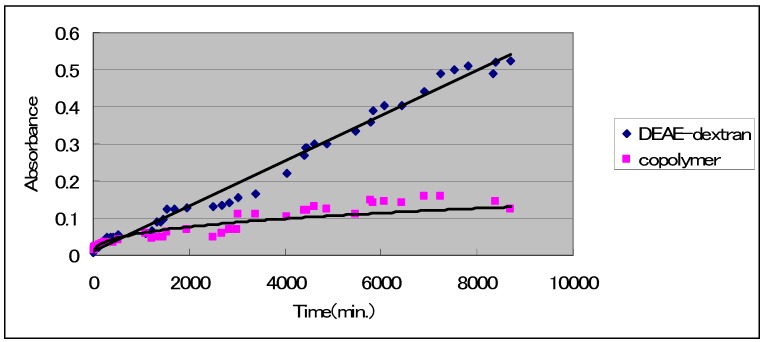
DNase degradation times for foreign DNA complex with DEAE-dextran-MMA graft copolymer and DEAE-dextran, respectively. DNase I degrades both double-stranded and single-stranded DNA endonucleolytically, producing 3´-OH oligonucleotides. Toluidine Blue (TB) is isolated in water from DNA after the degradation, as the DNA is stained with TB. This shows the absorbance of TB isolated from DNA in each sample in the water with a spectrophotometer.

As a result of confirming higher expression levels with DDMC (Figure 1), it was also thought that cellular toxicity decreased relative to DEAE-dextran. Thus, the protective effects of DNase were investigated *in vitro*. The effects of DNA stabilization have been understood based on differences in the protective effects of DNase. Specifically, based on the results (Figure 2), decomposition of DNA progressed from the start with DEAE-dextran/DNA, and a large quantity of toluidine blue was released, giving a significant change in absorption [12]. With DDMC/DNA, on the other hand, decomposition of DNA progressed slightly, and the change in absorption was extremely small. A significant difference was thus seen between DEAE-dextran and DDMC. The action of DDMC in protecting against DNase decomposition was dramatically increased in comparison to DEAE-dextran, and this is thought to be one of the causes of the increase in transfection efficiency.

### Complex Formation Reaction Mechanisms

The difference in protein expression due to DDMC and DEAE is thought to be caused by different complex formation reactions, particularly when their concentrations are very low. With the DNA and DDMC complex formation reactions, the hydrophobic bonding force is strongly influenced by the hydrophobicity of the grafted MMA regions, as well as the Coulomb forces and hydrogen bonding forces, thus giving rise to a reversible equilibrium relationship. The Michaelis-Menten complex formation reaction is thought to occur as follows:

Formed complex amount = K1 (DNA concentration) (DDMC concentration)
(1)

The amount of formed complex is proportional to the RLU value. The formation reaction for the complex between DEAE-dextran and DNA is nearly non-reversible because it depends mostly on Coulomb forces, and the reaction is first-order with respect to DEAE-dextran concentration. The reaction is thought to be expressed as follows:

Complex formation amount = K2 (DEAE-dextran concentration)
(2)

**Figure 3:**
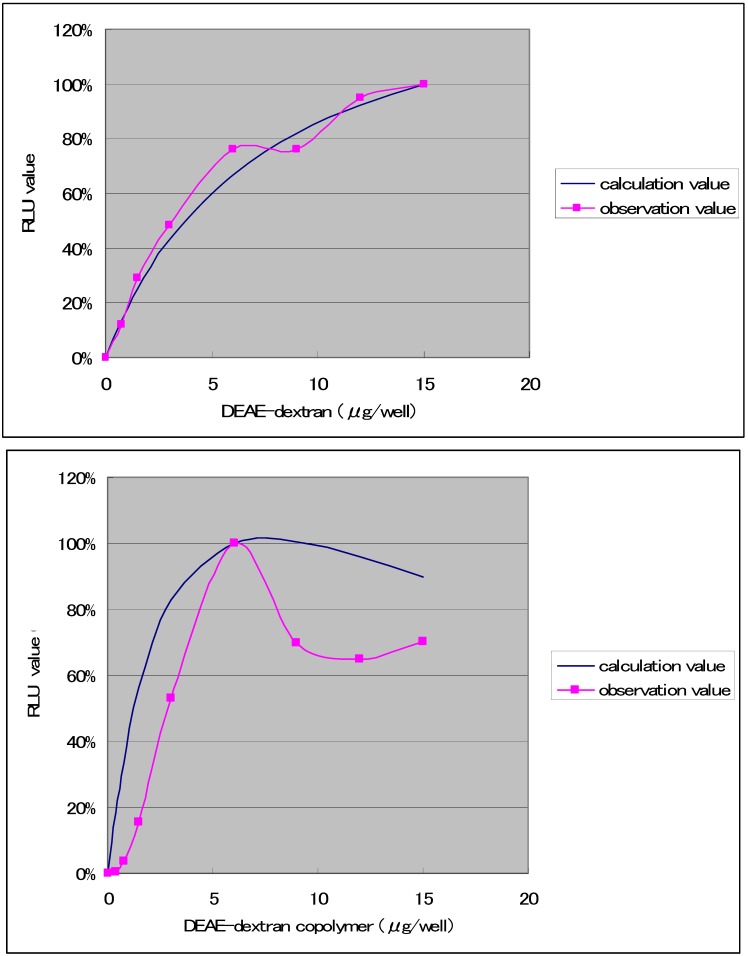
Transfection of COS-7 cells with samples of DEAE-dextran and DEAE-dextran-MMA graft copolymer having a grafting rate of 130% and including 0.075 μg of DNA. Maximum luciferase expression within each experiment was set at 100%.

The results (Figure 3) were obtained with regard to combinations that produced high transfection efficiencies when the transfection solution was diluted to 10.9 times, and the amount of DNA was held constant at 0.075 μg, while varying the amount of DDMC from 0 to 15 μg. With DEAE-dextran, it has been reported that the y/x ratio with respect to DNA (weight ratio) is optimally 1/50. Specifically, it has been reported that the optimal ratio of DEAE-dextran is about 50× relative to the amount of DNA [13]. In contrast to the results in Figure 1, the RLU value of DEAE-Dextran at this very low concentration exhibits higher values than DDMC. The RLU value is thought to be directly related to the potential for complex formation. The complex formation capacity is thought to give rise to a reversible equilibrium relationship, which can be expressed as a Michaelis-Menten equation:

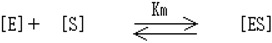

[E][S]/[ES] = Km
(3)

Normally, the relationship is between enzyme and substrate, but in this case, [E] is used to represent the concentration of DEAE-dextran or DDMC, and [S] is used to represent DNA concentration. Taking the initial DEAE-dextran or DDMC concentration as [E0], then:

[E] = [E0] - [ES]
(4)

Inserting these values, the complex concentration becomes:

[ES] = [E0][S]/(Km+[S])
(5)

With DDMC, the Coulomb forces are small (low affinity between E and S, and the fact that [S] is small has a direct influence on the complex formation). As Km increases, the complex becomes unstable, and [S] is negligible relative to Km. With this formula, assuming Km >> [S], the complex concentration becomes:

[ES] = [E0][S]/Km
(6)

This is the case for DDMC, and it is highly likely that the complex is strongly influenced by concentration conditions. In other words, it is thought that a very low DDMC concentration will have a significant influence on complex formation.

Conversely, considering DEAE-dextran, complex formation is stabilized when the Coulomb forces are large (high affinity between E and S, and the fact that [S] is small does not have a direct influence on the complex formation). As Km is small, Km thus conversely becomes negligible in comparison to [S]. Assuming that Km << [S], the complex concentration similarly becomes:

[ES] = [E0]
(7)

This indicates that complex formation is proportional to DEAE-dextran concentration. In other words, it is likely that there is no significant influence on a quantitative complex formation by DEAE-dextran concentration, even when the concentration is very low.

However, the Michaelis-Menten complex formation reaction between DDMC and DNA is thought to be significantly influenced by concentration. The relationship is expressed in Figure 3 using K1 = 1.055 × 10^-7^ (μg/well) and K2 = 1.626 × 10^-5^ (μg/well), respectively, as determined at the maximum RLU values, and normalizing the RLU values by taking the maximum experimental values as 100%. The figure shows a good correspondence with both DEAE-dextran and DDMC under conditions of 48 h and 0.075 μg of DNA. For the concentration represented on the horizontal axis, using 0.075 μg DNA and 0.75 μg DDMC, with a total volume of 30 μL for the D-MEM not containing FBS, the DNA concentration is 0.075 μg/30 μL, or 0.0025 μg/mL. The DDMC concentration is 0.75 μg/30 μL, or 0.025 μg/μL. Though the vertical axis in Figure 3 having RLU should be normally the amount of complex, because of this proportion in the amount of complex to RLU, the reaction mechanism may be understood to be analogous if the trend shown in the figure is similar. We found that 48 h is the optimal condition for DEAE-dextran. However, with the complex formation reaction for DEAE-dextran and DNA, Coulomb forces are well understood to be the primary factor in the Poly-ion complex (PIC) reaction, and thus experiments were carried out to compare the DDMC complex formation reaction with the case of DEAE-dextran [3,4]. 

Considering equation (4), in the transfection procedure, it is conceivable that that DNA and DDMC form a complex during incubation period for 2.5 h, or that they do not form a complex. Assuming that formation occurs, because D-MEM culture solution containing 10% FBS is used during this period, its influence on complex formation must be considered. It is also possible that pH has a strong influence on the complex formation reaction, and that FBS promotes DDMC decomposition. Based on these considerations, investigations concerning the use of culture solution not containing serum specific for transfection or PBS buffer will be required in the future. In addition, if there is unreacted compound that does not form complex, it is important to understand the degree to which this damages cells [14]. With DDMC, the incubation time was set at 2.5 h. Further investigations are necessary in order to determine the significance of this time period.

**Figure 4:**
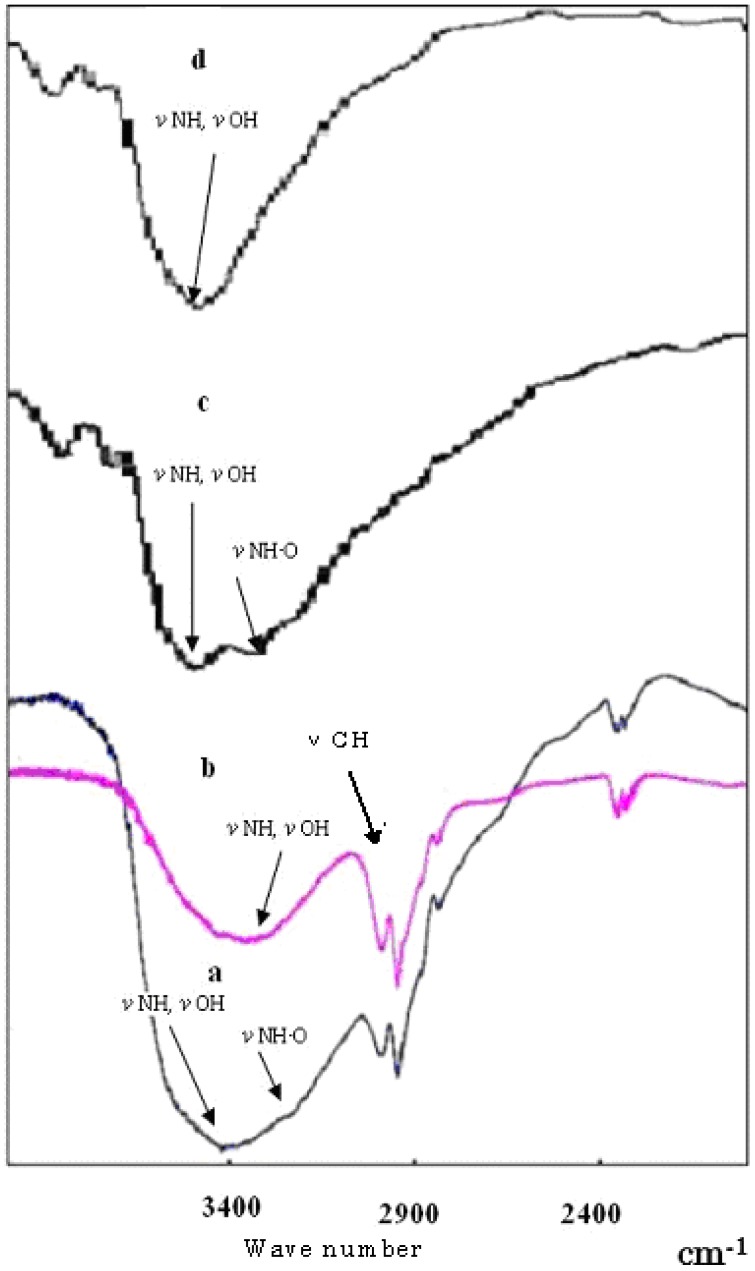
Infra-Red absorption spectra: a, complex of DDMC/DNA; b, DDMC; c, complex of DEAE-dextran/DNA; d, DEAE-dextran.

### Hydrophobic Bonding Contribution

The Michaelis-Menten equation is most suitable for biological reactions such as enzyme reactions in which hydrophobic bonding and hydrogen bonding participate in a complex manner. At present, simulations of DDMC complex formation reaction based on Michaelis-Menten equations have shown that DNA and DDMC complex formation involves a poly-ion complex, and a complex formation mechanism has been proposed in which hydrophobic bonding and hydrogen bonding participate in a complex manner. It is thought that the DNA is thereby condensed and protected from intracellular decomposition, and that this complex also facilitates passage through the nuclear membrane and into the nucleus. Figure 4 shows the infrared absorption spectrum in the vicinity of wavelengths 1,900 to 3,900 cm^-1^ for the complexes formed by reactions between DNA and DDMC (graft ratio 100%) or DEAE-dextran according to the procedures of Sections 2 and 4. With both DDMC and DEAE-dextran, the DNA complexes show hydrogen bond absorption due to the stretch vibrations of N-H, O-H and NH-O in the vicinity of 3400 cm^-1^ which are larger and broader than in the respective starting substances. In addition, N-H and O-H absorption have shifted to the high-energy side.

This means that interactions due to intramolecular hydrogen bonding itself are weak, and that the complexes are condensed. Although it was concluded that DDMC and DEAE-dextran with bonded DNA have decreased entropy relative to their unbonded states, this is to be expected based on stability with respect to stress. These results can be thought to be due to the occurrence of steric alterations for each. Of course, the high-energy shift is clearly larger for the DDMC/DNA complex. This intermolecular hydrogen bonding serves as a driving force for folding into a neat steric structure, and Figure 4 shows the absorption of the C-H stretch vibration in the vicinity of 3,000 cm^-1^ for DDMC, and this peak becomes broader with the DNA complex. The above results also demonstrate the presence of significant hydrophobic bonding in the DDMC/DNA complexes.

### Cell Transfection

The transfection efficiency into cells is said to be strongly dependent on DNA structure. DNA undergoes continuous coordinated changes from a swelled coil state to a condensed state (globule) when in solution, which is known as DNA condensation by a coil-globule transition, and the activity of the DNA changes from ON to OFF. This may induce discrete ON/OFF switching in transcriptional activity. From the standpoint of the transfection process, condensation must be understood as OFF. Specifically, when the DNA is in a compact closed state, the condition of DNA contraction shifts to favor passage through the cell membrane and DNA decomposition inside the cell [10]. At this stage, the important points are: 1) how the nucleic acid complex is efficiently taken into the cell [2]; 2) whether this suppresses decomposition of DNA in the cytoplasm or endoplasmic reticulum; 3) how to bring about efficient release from endoplasmic reticulum into cytoplasm; 4) how to bring about efficient transport from the cytoplasm to the nucleus; and 5) the intended state of the nucleic acid molecules for allowing transcription in the nucleus.

Transfection into cells, however, is thought to depend on endocytosis (phagocytosis), which in turn depends on DNA and DDMC complex formation, meaning that the complex formation conditions are critical. This Michaelis-Menten complex formation reaction is similar to actual complexes of DNA and histones in the body. With histone complexes, it is clear that DNA transcription depends on hydrophobic bonding alterations that are under control of acetyl groups. In our case, it was also thought that the hydrophobicity of DDMC has a strong influence on DNA transcription, depending on the environment.

**Figure 5:**
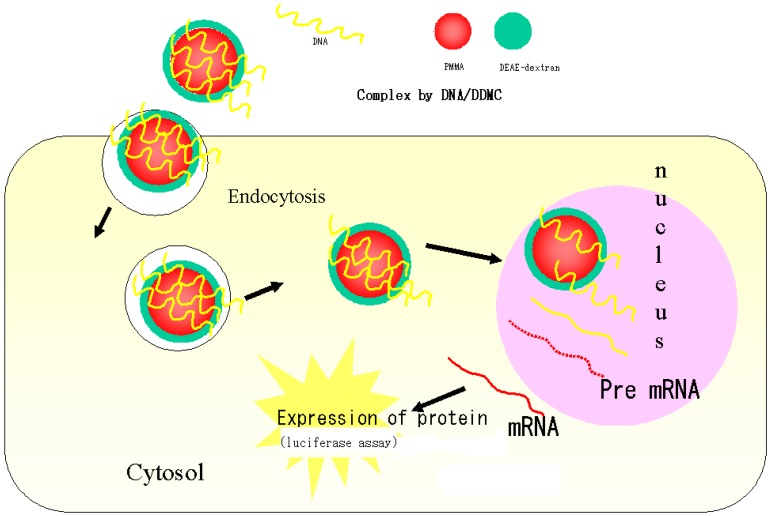
Schematic drawing of putative delivery pathways for foreign DNA complex with DEAE-dextran-MMA graft copolymer.

In addition, with cellular endocytosis, the PMMA portion, which is the hydrophobic domain of DDMC, is important for passage through the cell membrane. The DNA and DDMC complex formation reaction is strongly influenced by pH and charge ratio, but this reaction is one in which electrostatic bonding occurs via the minus charges of the phosphate esters of DNA and the plus charges of the DDMC, and the complex is thus is referred to as a poly-ion complex. Hydrophobic bonding and hydrogen bonding contribute to this in a complex manner, and the DNA is thus condensed and protected from decomposition inside the cell. It is also thought that this complex formation facilitates passage through the nuclear membrane to the nucleus [7]. Protection from decomposition in cells means protection from the action of DNase and dextransucrase in the cell, and it is thought that DDMC is superior to DEAE-dextran, which is constituted from PIC bonds (simple electrostatic bonds). However, the extent to which DDMC is introduced into cells is unclear, and future investigations are thus required. Figure 5 shows a schematic diagram of how DNA forms complexes with DDMC macromolecular micelles, how endocytosis occurs, and how the complex reaches the cell nucleus.

## Conclusions

DDMC, which is used as a carrier for gene introduction, can be sterilized by autoclaving, has better transfection efficiency relative to DEAE-dextran alone, and is also thought to have lower cellular toxicity. For these reasons, it is expected to be utilized with cells derived from arthropods in our future work, as well as cells derived from mammals.

## Experimental

### Reagents

The pGL3-Control Vector manufactured by Promega was used as DNA encoding luciferase, D-9885 (estimated molecular weight, Mw 500,000, hydrochloride) manufactured by Sigma-Aldrich Chemical was used for the DEAE-dextran, and the DDMC had a graft ratio of 130% and was used at concentrations of 10, 20 and 28.6 mg/mL. The Bright-Glo Luciferase Kit (Promega) was used for luciferase reagents, and GloLysis Buffer (Promega) was used as a cell lysis agent.

### DEAE-Dextran-MMA Graft Copolymer

The DEAE-dextran-MMA graft copolymer (DEAE-dextran-MMA graft copolymer; DDMC) is a tetravalent cerium salt of DEAE-dextran that had been graft polymerized with methyl methacrylate ester (MMA) [1]. The copolymer had DEAE-dextran as the backbone polymer with PMMA as a branch polymer. A structure having a hydrophilic–hydrophobic microseparated domain was thus formed with the DEAE-dextran parts as hydrophilic domains and the branch polymer PMMA parts as hydrophobic domains.

*Definition of Copolymer Graft Ratio:* The grafting ratio is defined as the PMMA weight (branch polymer)/DEAE-dextran weight (backbone polymer). With the DDMC graft reaction used in the experiments, the PMMA (branch polymer)/DEAE-dextran (backbone polymer) graft ratio was 2.6 g/2 g, or 130%, when the grafting reaction progressed to completion.

*Definition of Charge Ratio:* The P/N ratio is defined as the charge ratio. With the complex formation reaction between DDMC (N: 1.4%) and DNA (P: 5.3%), the compound formed by ionic bonding (poly-ion complex; PIC) is the primary constituent element, and thus the constituent ratio is expressed as the weight ratio along with the charge ratio.

P/N (charge ratio) = (y × 0.053 × 14)/(x × 0.014 × 31)

DNA/DDMC = y/x (weight ratio); P: 5.3%; N: 1.4%; P atomic weight: 14; N atomic weight: 31; y: amount of DNA; x: amount of DDMC

### Cell Transformation

*Test Cells:* COS-7 African green monkey kidney cells were used (CV-1 monkey cells transformed with SV40 having a replication initiation point defect; expressing the SV40 large T antigen).

*Calculation of Cell Number*: A glass pipette was used to remove medium from a 75-cm^2^ flask (Corning) containing COS-7 cells cultured in D-MEM medium containing 10% FBS. Next, 1× PBS(-) solution was introduced into a 6-mL flask, the surfaces of the cells were washed, and the 1× PBS(-) solution was removed. This procedure was carried out twice. Next, 3 mL of 2× 1% trypsin/EDTA solution was added to release the cells, and 12 mL of D-MEM medium containing 10% FBS was then added. Cells were thoroughly pipetted, and 750 μL of 1× PBS(-) solution and 200 μL of 0.5% Trypan Blue were immediately added to 50 μL of the COS-7 cells that had been suspended. The cells were thoroughly agitated, and only live cells were counted using a hemocytometer. A 96-well microtiter plate was used, and the cells were introduced at a cell count of 2 × 10^4^/well. To prevent drying, 100 μL/well of D-MEM medium was added to the empty wells in the microtiter plate. Subsequently, the microtiter plate containing COS-7 cells was cultured for one day under conditions of 37 °C and 5% CO_2_.

*Production of Transfection Solutions:* Plasmid DNA (0.05 μg; pGL3-Control Vector) encoding luciferase was diluted with 2.6 μL of 1× PBS (-) solution in a sterile tube, and 0.14 μL of DEAE-dextran or DDMC were each added and stirred thoroughly to prepare solutions, respectively.

*Transfection Method:* COS-7 cells were cultured overnight, and the culture solution was removed from each well of the 96-well microtiter plate. Next, cells were washed twice with 100 μL of 1 × PBS (-) solution, and 2.79 μL of transfection solution was added to the COS-7 cells in each well. The 96-well microtiter plate was gently but sufficiently agitated during culture so that the solution was well circulated. The microtiter plate was incubated for 30 min at 37 °C while swirling every 5 min during culture. Subsequently, 28.8 μL/well D-MEM medium containing 10% FBS was added to the microtiter plate during culture, and the plate was incubated for 2.5 h at 37 °C. Subsequently, the D-MEM medium was removed, and 100 μL of fresh D-MEM medium containing 10% FBS was added and incubated for 24 to 96 h at 37 °C.

*Emission Measurement:* After 24 h (or 48, 72, or 96 h), the plate containing the incubated COS-7 cells (including transfection solution) was removed from the incubator, the medium was removed, and the cells were rinsed with 50 μL/well of 1× PBS(-) solution. Next, 25 μL/well Glo Lysis Buffer was added, and the culture plate was swirled. After 5 min, 25 μL/well Bright-Glo Luciferase reagent was added, and emission measurements were carried out after 2 min using a SPECTRA Fluor Plus (Tekan) and LS-PLATEmanager 2001 (Wako Pure Chemical) to obtain RLU values. The measurement conditions were set to a gain of 150 and maximum integration time.

*Calculation of RLU Values:* In calculating the RLU values of the samples, two wells of the 96-well microtiter plate were used, and the average of the RLU values obtained from two parallel sample runs was determined.

*Sterilization Agents:* Antibiotic or antifungal agents were not added to the D-MEM medium containing 10% FBS used in culturing the cells. However, at the point when incubation for 2.5 h was completed after adding transfection solution, D-MEM medium containing 10% FBS and antibiotic-antifungal agent (penicillin/streptomycin/amphotericin B, manufactured by Invitrogen) was used for the culture solution.

*DNase Decomposition Testing:* A 1-mL sample of DNA solution (10 mg/mL) derived from salmon sperm and 1 mL of 0.005% toluidine blue solution (pH 7) were allowed to react, after which 1 mL of DEAE-dextran solution (10 mg/mL) or 1 mL of DDMC having an equivalent charge (28.6 mg/mL) was added and allowed to react, thus causing deposition as PIC complex. The solutions were allowed to pass through #5 filter paper (Advantech), and this material was then transferred into a test tube for each filter paper. Next, 4 mL of distilled water was added, followed by 0.01 mL (0.01 mg) of RQ1 RNase-Free DNase and 0.1 mL of 10× PBS(-) buffer solution. DNA decomposition was allowed to occur at 30 °C. The absorption of toluidine blue released into the supernatant liquid as a result of this series of reactions was then measured at 633 nm.
